# IMPROVE 1.0: Individual Monitoring of Psoriasis Activity by Regular Online App Questionnaires and Outpatient Visits

**DOI:** 10.3389/fmed.2021.648233

**Published:** 2021-06-22

**Authors:** Natalie Garzorz-Stark, Sarah Beicht, Veronika Baghin, Sebastian P. Stark, Tilo Biedermann, Felix Lauffer

**Affiliations:** ^1^Department of Dermatology and Allergy, Technical University of Munich, Munich, Germany; ^2^Division of Dermatology and Venereology, Department of Medicine Solna, Center for Molecular Medicine, Karolinska Institutet, Stockholm, Sweden

**Keywords:** smartphone, application, psoriasis, inflammatory skin disease, personalized medicine

## Abstract

Smartphone apps gain more and more importance in supporting management of chronic diseases. Psoriasis is a highly prevalent, lifelong chronic inflammatory skin disease with a high impact on patient's quality of life. Disease management includes regular topical and systemic treatment of skin lesions as well as co-treatment of metabolic and psychologic disorders. In this study, we investigated the potential of a new smartphone app (IMPROVE 1.0) for individual monitoring of disease activity and disease influencing factors. Twelve out of 50 psoriasis patients asked for study participation performed self-assessment of psoriasis severity, life quality, and stress scores using the app over a period of 1 year. Every 2 months, study participants were carefully examined by a dermatologist in order to control the quality of app-reported data. We found that psoriasis severity and life quality values as entered in the app closely correlate to physician's examination. Furthermore, we detected strong correlations of disease activity with life quality and psoriasis serum biomarker. Temporal relations between psoriasis aggravation and previous changes of lifestyle factors, such as increased stress levels, were observed in individual patients, indicating a high potential for preventive interventions in future psoriasis apps. The vast majority of study participants evaluated IMPROVE 1.0 app positively and wish to include the app into their daily life. Hence, we demonstrate that smartphone apps are a useful tool to raise self-awareness for the dimensions of complex diseases and fully integrate psoriasis patients into individual disease management. These data are important to develop more advanced digital tools supporting the management of chronic diseases in the future.

## Introduction

Smartphone apps play an increasingly important part in daily life. They are easily accessible, are handy, and facilitate access to information, communication, or management of routine tasks such as banking. As health care systems are limited by financial restrictions and personal capacities, smartphone apps might more and more support or take over routine physician's work. Several digital health tools have been developed over the last decade (1). While a plethora of smartphone apps are created for patient education and mainly deliver information in a more convenient way (2, 3), the major restriction of more advanced apps is the lack of objective measurement tools to perform long-term disease monitoring. Therefore, many apps are linked to a conventional medical device in order to assess, for example, blood pressure, blood glucose, or overall healthy lifestyle in a prospective way (4, 5). However, to establish an app in a larger cohort of patients, all essential tools must be executable by the patients themselves without any further technical devices. For this purpose, inflammatory skin diseases (ISD) are an ideal disease model, as disease activity is usually assessed by morphological scores, such as the psoriasis area and severity index (PASI).

Psoriasis is one of the most common ISD, which affects up to 3% of the general population in western countries (6). Patient's life quality if critically diminished by visible and stigmatizing plaques of inflamed skin, itch, scaling, and comorbidities (7, 8). In the latter case, the metabolic syndrome, addictive diseases, and psychological disorders are the major causes of morbidity and mortality (7). However, obesity or distress can—vice versa—also impair psoriasis severity (9). Therefore, psoriasis patients need a disease management considering individual disease and lifestyle factors (10)—a comprehensive approach that might profit from digital support by a smartphone app. However, it is so far unknown if patients are capable to reliably judge psoriasis severity and reflect about comorbidities using an app and if they appreciate a digital tool for their individual disease management.

To test whether apps are useful to monitor psoriasis disease activity, we created a new app (IMPROVE 1.0) and asked psoriasis patients to regularly enter self-PASI, life quality, and stress questionnaires as well as lifestyle factors over a period of 12 months. In a prospective study, we compared data collected by the app with physician's examination, with disease scoring performed by dermatologists and biomarkers. Furthermore, we collected patients' feedback on utility and helpfulness of IMPROVE 1.0 in order to gain further insight into the potential of a psoriasis app in clinical practice.

## Methods

### Study Cohort

**Figure 1:**
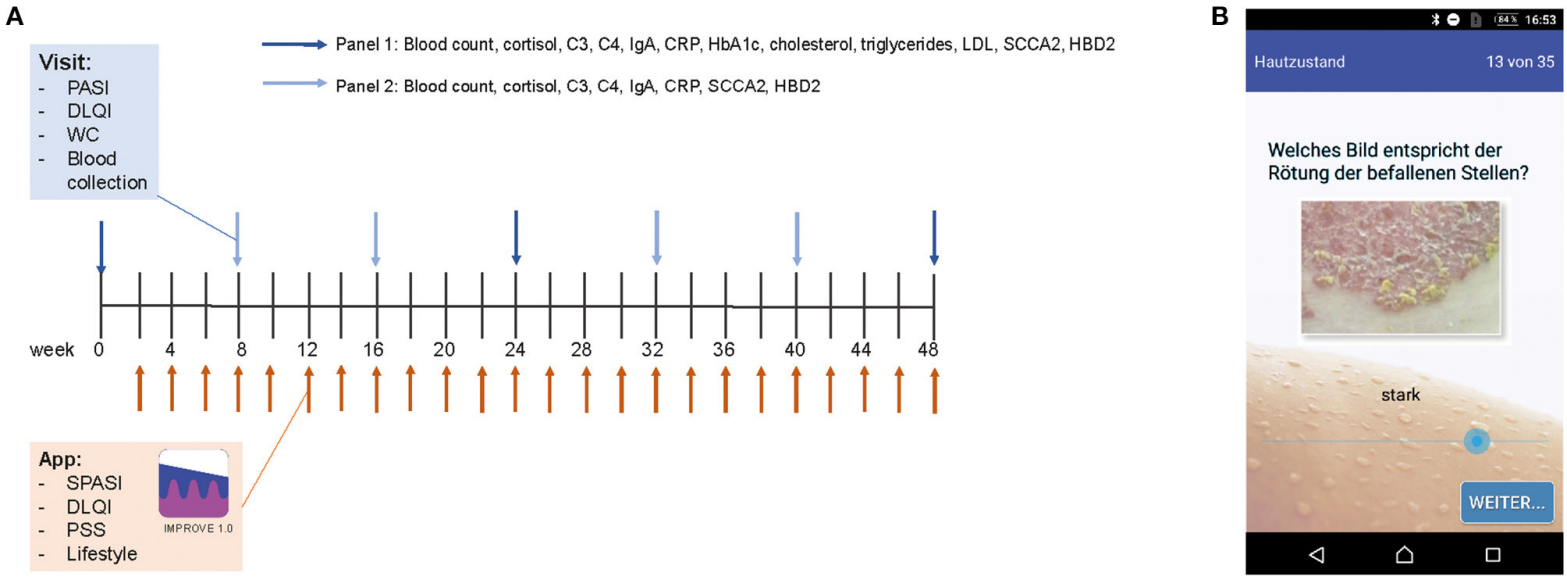
Study design and app. PASI, DLQI, and waist circumference (WC) of psoriasis patients were assessed every 8 weeks. Besides, blood was taken and examined for blood counts and serum parameters (Panel 1 at visits 2, 3, 5, and 6 and Panel 2 at visits 1, 4, and 7) **(A)**. Biweekly, patients filled out an app questionnaire including SPASI, DLQI, PSS, and questions on lifestyle factors. Assessment of disease severity was supported visually by example pictures of psoriasis for redness, thickness, and scaling (here exemplarily shown for redness) **(B)**.

Fifty adult patients (age ≥ 18 years) with histologically confirmed diagnosis of psoriasis regularly seen at our outpatient clinic who used a smartphone and had experience with smartphone apps were asked to participate in the study. Reasons for not participating were lack of time and/or no smartphone with Android system (app only programmed for Android system) at patient's disposal. Thirteen of these patients were enrolled, and 12 of the enrolled patients completed the 48-week study. One patient did not continue the study after the first outpatient visit for personal reasons. Patients were given an introduction of the app and every 2 weeks ± 1 week; they filled in the smartphone app questionnaire assessing disease severity and lifestyle factors (see below). Lifestyle factors and their potentially aggravating effect on psoriasis were explained at the beginning of the study to each patient but no intervention with regard to lifestyle factors was initiated. Every 8 ± 2 weeks, patients were examined by a physician during an outpatient visit. Moreover, blood samples were taken at each visit and analyzed for serum markers and blood count (see Figure 1A). At the end of the study, patients completed a final evaluation questionnaire on the app and the study. The study was approved by the Ethics Committee of the Faculty of Medicine of the Technical University of Munich (63/16S) and written informed consent was obtained from the patients.

### Smartphone App Improve 1.0

The app was programmed using Eclipse for androids, an IDE for developers creating Android applications (http://www.eclipse.org). The app was sent to the patient *via* e-mail and downloaded by the patient. An introduction to the app was given by the study team. The app consisted of the DLQI questionnaire to measure the quality of life (11), the PSS questionnaire to measure the perception of stress (12), and of questions on their lifestyle (consumption of alcohol, cigarettes, and fast food; frequency of doing sports; body weight; and sleep), which were assessed on a verbal rating scale that was converted to a numeric rating scale for analysis (0–4, except for the amount of cigarettes 0–5). Besides, the patients assessed the severity of psoriasis according to the simplified PASI (SPASI) (13). The area of involvement (area score) was assessed on a 0–6 scale [0: no lesions, 1: ≤10 palms of the hand (PTH), 2: ≤30 PTH, 3: ≤50 PTH, 4: ≤70 PTH, 5: ≤90 PTH, and 6: complete body affected]. The average redness (R), thickness (T), and scaliness (S) of the lesions were assessed using a verbal rating scale that was converted to a numeric rating scale for analysis (0–4 scale). To facilitate this assessment, each scale point was visualized by a representative picture of the severity (see Figure 1B). SPASI was calculated as follows: SPASI = Area score × (R + T + S).

### Blood Parameters

Blood counts (leukocytes, lymphocytes, neutrophils, eosinophils, basophils, and monocytes) and serum proteins (Cortisol, C3, C4, IgA, CRP, HbA1c, cholesterol, triglycerides, and LDL) were assessed by clinical routine laboratory. ELISA for human squamous cell carcinoma antigen 2 (SCCA2; PeproTech, Germany) and Beta-defensin-2 protein (HBD2; MyBioSource, CA, USA) were performed according to the manufacturer's instructions.

### Statistical Analysis

Statistical analysis was performed using GraphPad Prism 7.00 Software (GraphPad Software, La Jolla, CA). Two-tailed paired sample test was used to compare variables of two different time points (beginning vs. end of the study) or of two different evaluators (mean PASI or DLQI as assessed by the physician vs. mean SPASI or DLQI as assessed by the patient *via* app) within individuals. Variables were correlated using Pearson correlation. Average Pearson coefficients across patients were obtained by transforming each correlation coefficient using Fisher's *z*, calculation of the mean of the *z* values, and back-transformation of the mean *z* value to the mean correlation coefficient. Parameters of outpatient visits were correlated with parameters assessed by the app if data of both visits and app were available from the same week. Correlation matrix was built using the agglomerative clustering tool within the scikit-learn package of Python 0.23.2 (https://scikit-learn.org/stable/). Mean values are shown with standard error of the mean (SEM).

## Results

### Design of the Study and Characterization of the Study Cohort

Twelve psoriasis patients completed the 48-week study consisting of biweekly online smartphone app questionnaires and clinical outpatient visits every 8 weeks including clinical examination and blood tests (see Figures 1A,B). Details on the study cohort are listed in Table 1. Within the study cohort, the mean age of patients was 43.25 ± 4.11 years and 67.7% of the participants were male. Mean age of onset of psoriasis was 20.92 ± 3.77 years, and PASI at the time of the individual start with the study (week 0) was 7.37 ± 1.79. Fifty percent of participants were smoker, mean BMI at week 0 was 28.38 ± 1.68, and 8 out of 12 patients suffered from comorbidities such as type 2 diabetes and obesity.

**Table 1:** Clinical characteristics of the patients included in the study.

**Patient (no.)**	**Sex**	**Age**	**Psoriasis therapy (week of study: 0–48)**	**Comorbidities**	**Age of onset of psoriasis**	**Course of disease**	**Smoker**	**PASI at week 0**	**BMI at week 0**
1	M	33	Secukinumab (0–56)	Overweight	20	Stable with no symptom-free intervals	No	0.5	28.4
2	F	54	Methotrexate (0–23), none (23–37), topical glucocorticosteroids (37–48)	Type 2 diabetes, obesity, COPD, hypothyroidism, myocardial infarction at age 49	20	Progression	No (until age 49)	18	38.3
3	M	26	Ustekinumab (0–47)		0	Progression	No	10.7	25.9
4	F	42	Secukinumab (0–42), ixekizumab (42–48)	Obesity	20	Stable with no symptom-free intervals	No	3.6	29.7
5	F	27	Methotrexate (36–48), topical glucocorticosteroids (0–48)	Atopic dermatitis, allergic rhinoconjunctivitis	9	Progression	Yes	0.9	23.9
6	M	36	Fumaric acid (0–31), methotrexate (31–48)	Allergic rhinoconjunctivitis, migraine, arterial hypertension, overweight	33	Progression	No	12.6	29.8
7	M	32	Fumaric acid (0–4), topical glucocorticosteroids (0–52)		31	Progression	Yes	10.2	22.8
8	F	56	Topical glucocorticosteroids (0–4)		4	No symptoms age 12–35, thereafter progression	No	16	20.9
9	M	57	Secukinumab (0–44)	Obesity, prediabetes	36	Stable with symptom-free interval at age 52–57 years	Yes	1	30.7
10	M	45	Methotrexate (0–18 and 31–48)	Obesity, prediabetes	43	Progression	Yes	6.8	38.5
11	M	73	Fumaric acid (0–4), apremilast (4–23), secukinumab (23–48)	Type 2 diabetes, obesity	21	Stable with symptom-free interval at age 21–56 years	Yes	1.2	30
12	M	38	Apremilast (0–48)		14	Stable with no symptom-free intervals	Yes	6.9	21.67

### App-Reported PASI and DLQI Correlate Closely to Physician's Objective Assessment

**Figure 2:**
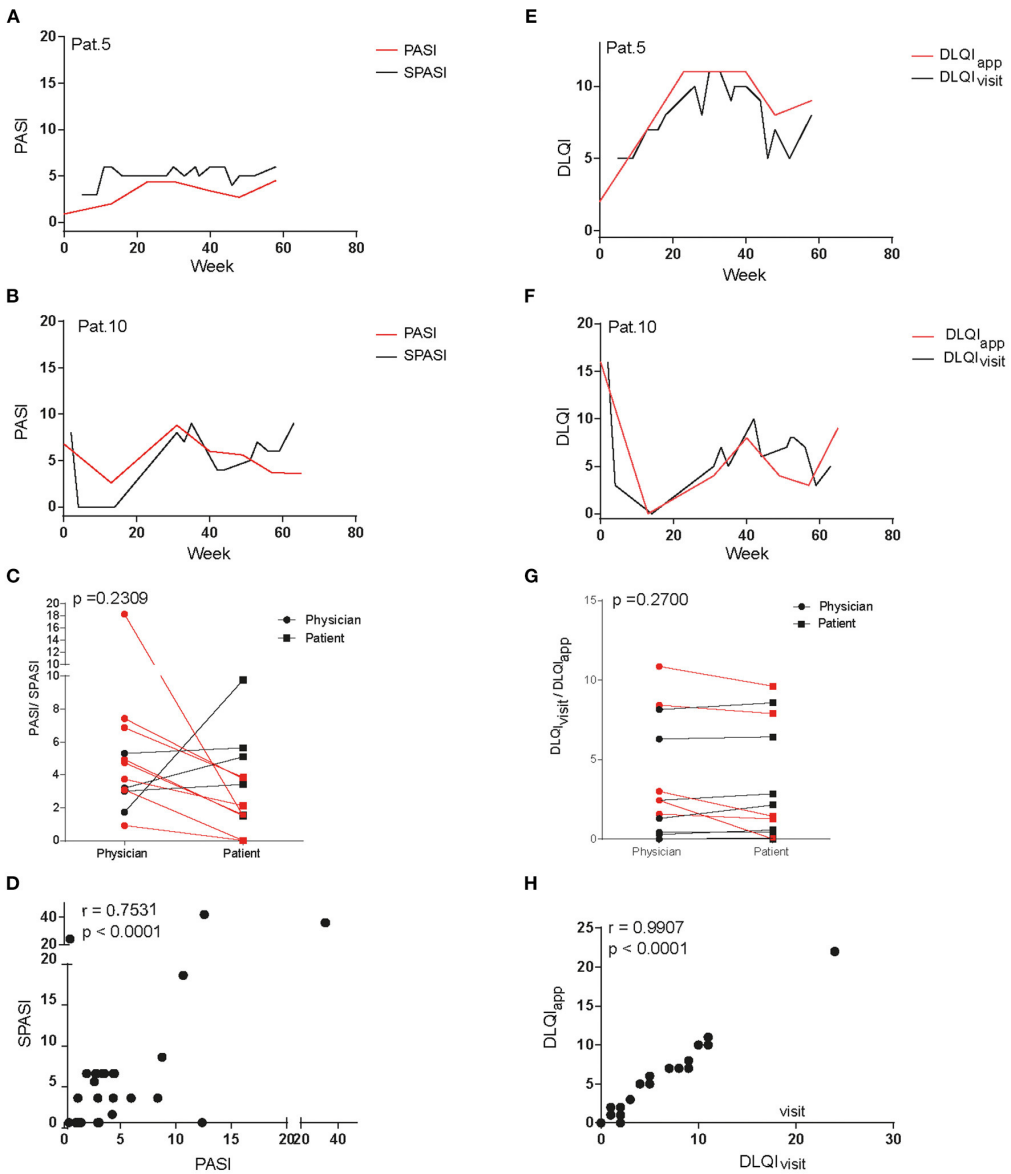
Comparison of dermatology life quality index and disease severity assessed at outpatient visits and *via* app questionnaire. Curves of PASI and SPASI take a similar course as exemplarily shown for patients 5 **(A)** and 10 **(B)**. Comparison of mean PASI values as measured by physicians and mean SPASI values as measured by patients during the course of the study show no difference on patient level **(C)**. SPASI and PASI across patients show high correlation **(D)**. Also, curves of DLQI_app_ and DLQI_visit_ take a similar course in patients 5 **(E)** and 10 **(F)**. Mean DLQI_visit_ values as stated during outpatient visits and mean DLQI_app_ values as determined via app during the course of the study do not differ on patient level **(G)**. DLQI_app_ and DLQI_visit_ across patients show high correlation **(H)**.

To assess if patients' reported disease activity would be in line with the physician's objective scoring of disease activity, curves for both SPASI as reported *via* app and PASI as determined by the physician during the outpatient visits were plotted together and are exemplarily shown for two patients (Figures 2A,B). Indeed, both curves showed a similar course (Figures 2A,B; other patients, Supplementary Figure 1) and mean PASI and SPASI did not significantly differ (*p* = 0.2409): Eight out of the 12 patients evaluated their PASI lower as the physician did, as shown by the mean over all PASI and SPASI values, respectively, over the course of the study (Figure 2C). However, SPASI and PASI values over all patients showed significant correlation (*r* = 0.75, *p* < 0.0001, Figure 2D). We next sought to compare DLQI as determined *via* app (DLQI_app_) with DLQI values given by the patient during the outpatient visit (DLQI_visit_). Also, here, both curves showed high accordance (**Figures E,F**; other patients, Supplementary Figure 2) and DLQI determined during visits and *via* app did not significantly differ (*p* = 0.27): 7 out of 12 patients reported lower quality of life *via* app than during outpatient visits as shown by the mean of all DLQI_app_ and DLQI_visit_ values, respectively, during the study (Figure 2G). Also, for DLQI, high correlation of DLQI_app_ and DLQI_visit_ over all patients could be found (*r* = 0.997, *p* < 0.0001, Figure 2E).

### DLQI and HBD2 Correlate With Disease Activity, While Stress Shows Individual Temporal Correlation With PASI

**Figure 3:**
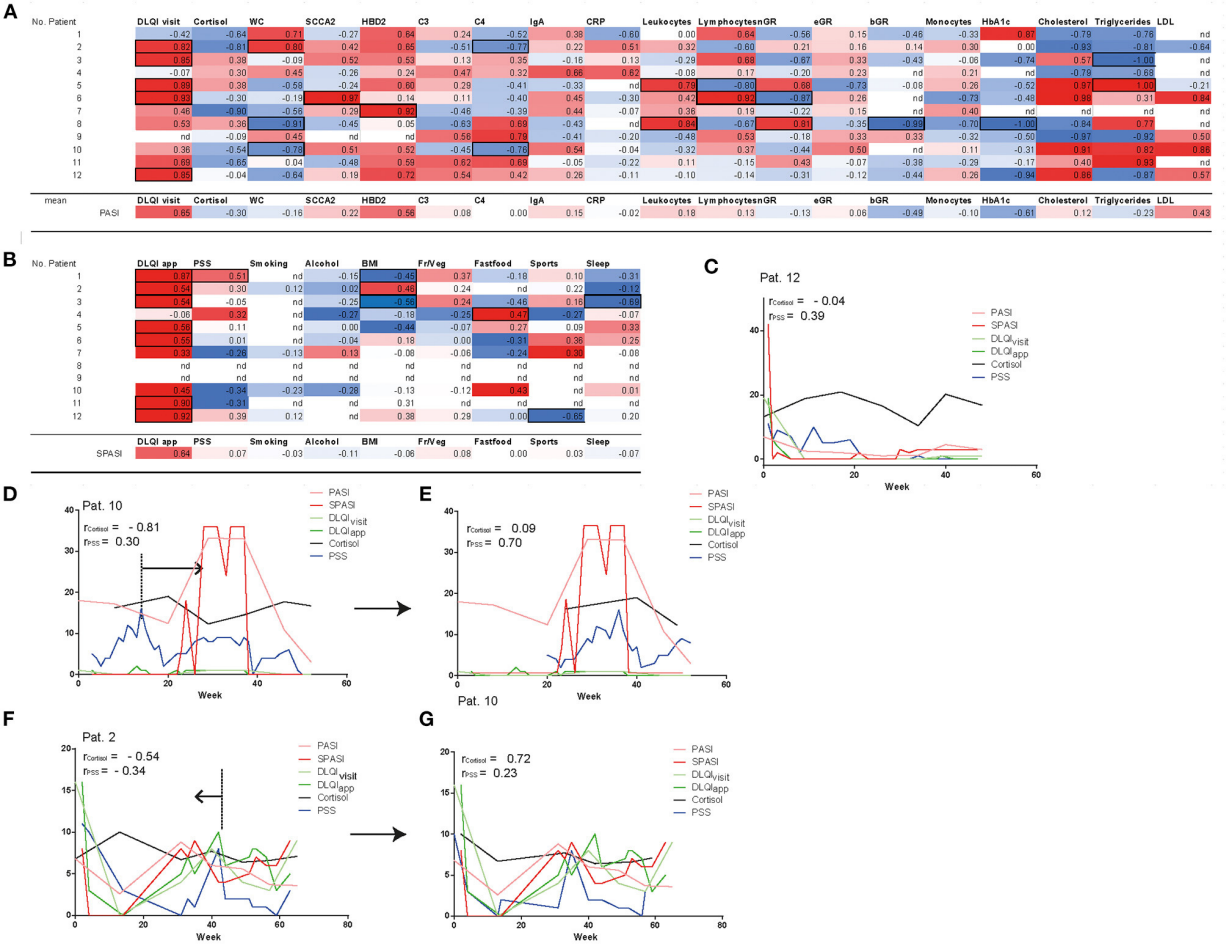
Correlation of disease severity with parameters assessed at outpatient visits and *via* app. PASI shows correlation with DLQI_visit_ and HBD2 **(A)**, SPASI with DLQI_app_ **(B)**. Significance of parameters (*p* < 0.01) on patient level are shown in black frames. Besides, mean correlation coefficients of all patients are shown for each parameter with PASI/SPASI. Nd = no correlation could be calculated. Fr/Veg = consumption of fruits and vegetables. Curves of cortisol, PSS, DLQI_visit_, DLQI_app_, PASI, and SPASI are exemplarily shown in patient 12 **(C)**, patient 10 **(D,E)**, and patient 2 **(F,G)** and reveal temporal correlation of stress and PASI. In patients 10 and 2, curves of PSS and cortisol are timely shifted **(E,G)** to reveal PSS and cortisol as factors temporally preceding **(D)** or succeeding **(F)** increase of PASI. nGR, neutrophils, eGR, eosinophils; bGR, basophils; Fr/Veg, consumption of fruits and vegetables.

To next assess factors correlating with PASI and SPASI on individual patient level but also across patients, we correlated PASI with serum parameters and DLQI_visit_. Besides, SPASI was correlated with DLQI_app_, BMI, stress, and lifestyle factors (see Figures 3A,B). Across all patients, PASI (SPASI) showed a mean correlation coefficient of 0.65 (0.64) with DLQI_visit_ (DLQI_app_). Moreover, human beta defensin 2 (HBD2), a previously described serum marker for psoriasis disease severity, showed the highest correlation coefficient among all measured blood parameters with disease activity (*r* = 0.56), which objectively confirmed correct assessment of PASI and SPASI in our cohort. Percentage of basophil granulocytes and levels of HbA1c showed a trend toward negative correlation with PASI. Interestingly, none of the lifestyle factors such as sleep, smoking, and consumption of fruits or vegetables correlated with SPASI across all patients. Instead, correlations on individual patient level between PASI and assessed parameters could be found (Figures 3A,B and Supplementary Figures 3A,B): For example, in patient 2, waist circumference (WC) and BMI significantly correlated with PASI and SPASI, respectively, whereas in patients 5 and 8, inflammatory activity is reflected on serum level by positive correlations between PASI and levels of leukocytes and neutrophils in the blood. In patient 3, e.g., amount of night sleep negatively correlated with SPASI. As stress has been discussed as a potential aggravator of psoriasis disease activity (14, 15), we next sought to investigate the influence of stress on PASI, SPASI, cortisol levels, and DLQI. Exemplarily shown for patient 12, a significant correlation between SPASI and DLQI_app_ (*r* = 0.91, *p* < 0.0001) and also for PASI and DLQI_visit_ (*r* = 0.85, *p* < 0.015) was found. No correlation of PASI with cortisol levels was observed; however, a trend for weak correlation with stress as measured by the PSS (*r* = 0.39, *p* = 0.077, Figure 3C) was observed, indicating that decrease of stress levels was timely accompanied by decrease in disease activity. In patient 10, however, one may come up with the hypothesis that rising cortisol and PSS levels preceded the increase of PASI (Figure 3D). Indeed, when shifting cortisol and PSS curves 20 weeks ahead, correlation values between SPASI and PSS increased from *r* = 0.3 and *p* = 0.07 to *r* = 0.7 and *p* = 0.0004 (Figure 3E). Increase in cortisol and PSS may also follow increasing PASI, as exemplified in patient 2 (Figure 3F): Indeed, when shifting both cortisol and PSS curves 10 weeks back, correlation values, though not at significant level, become positive for PSS and SPASI (PASI and cortisol) from *r* = −0.34 and *p* = 0.23 to *r* = 0.23 and *p* = 0.6 (from *r* = −0.54, *p* = 0.2146 to *r* = 0.72, *p* = 0.1671) (Figure 3G). Interestingly, PSS did not correlate with DLQI (*r* = 0.12) and cortisol levels (*r* = −0.31) across all patients (see Supplementary Figure 3C).

### Patients Reported Benefits of the Smartphone App

**Figure 4:**
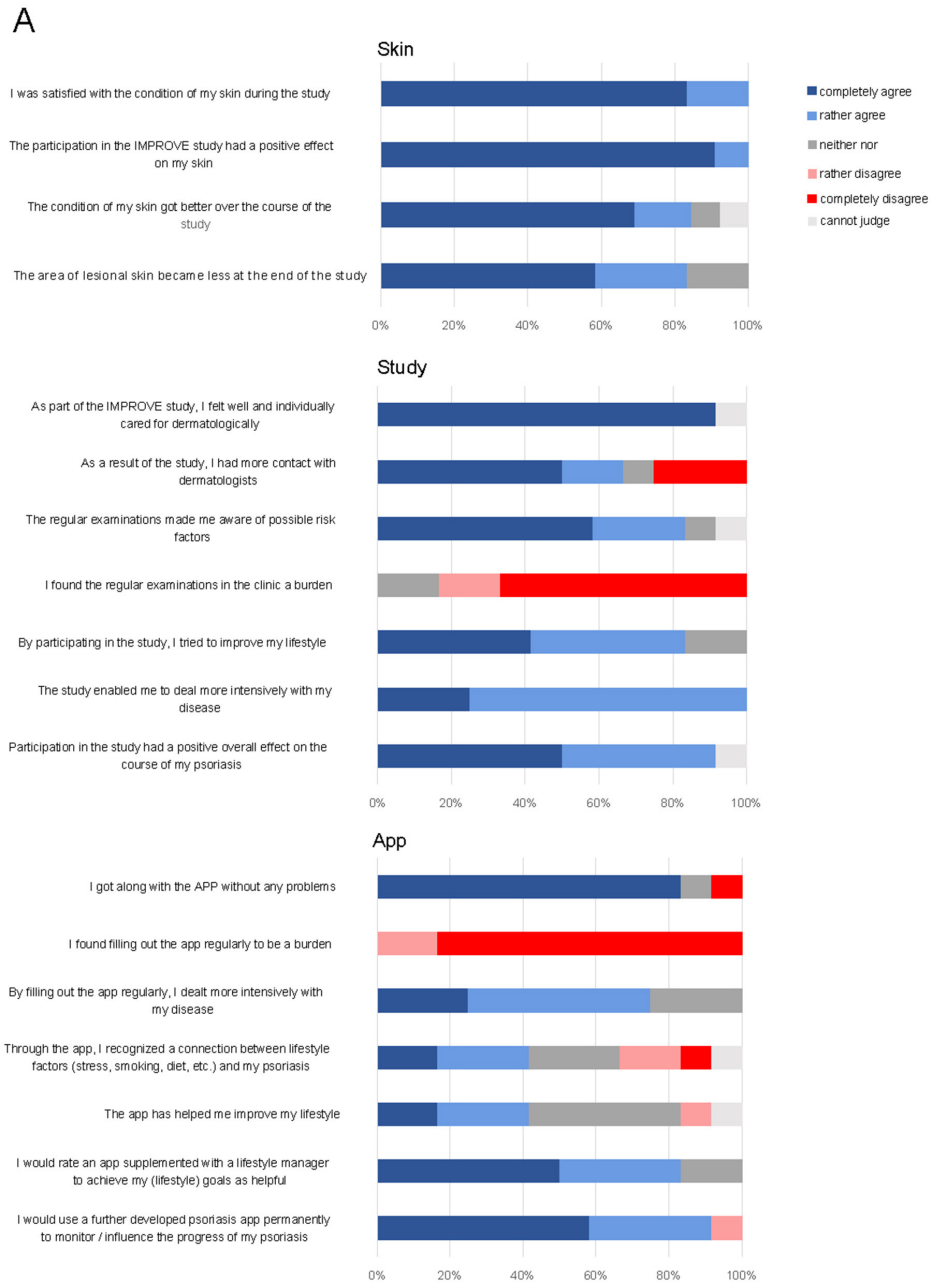
Final questionnaire at the end of the study on skin, study design, and app. Shown are patients' responses in percent on a verbal rating scale.

At the end of the study, patients were asked to evaluate the study regarding the skin condition, the study design and the app. More than 80% of the participants considered the study to have had a beneficial effect on their skin lesions (Figure 4); in particular, the individual care was highly appreciated. Indeed, PASI significantly decreased across all patients (7.37 ± 1.78 at the beginning of the study vs. 2.58 ± 0.45 at the end of the study, *p* = 0.0249, Supplementary Figure 4A). Only 3 out of 12 patients dealt more intensively with the disease by filling out the app questionnaire. This is reflected by the fact that none of the lifestyle factors (BMI, sports, consumption of fruits and vegetables, alcohol, and smoking) showed significant differences at the beginning of the study as compared to the end of the study (Supplementary Figures 4B–F). In contrast, consumption of fast food even increased (*p* = 0.0281). Fifty percent of patients would appreciate an updated version of the app supplemented with a lifestyle manager to achieve individual lifestyle goals.

## Discussion

In this study, we investigated the potential of a new smartphone app for assessment of psoriasis severity, life quality, and lifestyle factors. We found that patient reported disease severity (SPASI) using the app closely resembles PASI scoring performed by a dermatologist. Furthermore, we demonstrate that both PASI and SPASI significantly correlate to life quality (DLQI) and the well-established serum biomarker HBD2 (16). More than 80% of study participants gave a positive evaluation of the smartphone app, and mean PASI values significantly decreased during the entire course of the study.

Psoriasis is a lifelong inflammatory disease, which requires a comprehensive therapeutic concept. Patients need to be treated by several medical disciplines, for example, dermatologists primarily evaluating skin lesions or general physicians caring about metabolic disorders. Often, interdisciplinary communication, if any, relies on written reports and intuitively every physician involved mainly focuses on his own specialty, a situation that can be ineffective and time-consuming for both patients and physicians. Often, there is an active–passive relationship between health care professionals and patients, with patients being in a passive and inanimate role (17). Here, a smartphone app used by the patients would help to bring the patient back into the center of disease management as it was already shown for, e.g., neurological disorders (18). Regular assessment of the disease activity by the patients and easy display of disease course and disease-related factors *via* the app would clearly facilitate doctor–patient communication. Saving time of a detailed medical history allows more time for individual medical consultation. Furthermore, a structured evaluation of all disease-related factors in a smartphone app raises awareness and reflection about disease dimensions for health care professionals of different disciplines and patients.

Several apps supporting patients with chronic diseases or cancer have already been developed (19–21). In case of psoriasis in two randomized controlled trials, Sevendsen et al. demonstrated that a smartphone app improved patients' adherence to topical treatment and Spencer et al. showed that psoriasis patients learned more about their disease when using an app than a control group without digital support (22, 23). In contrast to these studies, IMPROVE 1.0 aims at not only facing one aspect of psoriasis, but on monitoring all disease-related factors. We did not choose a randomized, placebo-controlled study design, as the primary aim of the study was to compare data reliability between app and physician's examination in the same cohort of patients. Here, we could prove that data collected by the app, such as SPASI, correlate well to physician's scoring. Based on these findings, the next step of development will be to integrate more app functions. While PASI values decreased significantly during the study period, there was no change of lifestyle factors in our cohort, since no intervention for modulation of possibly aggravating lifestyle factors was initiated. However, we observed in individual patients that a change in BMI or stress level during the 12-month observation period was followed by an increase of PASI, indicating that our app can detect lifestyle changes preceding a shift in PASI. Thus, a valuable feature of an updated app version (IMPROVE 2.0) would be an implementation of an “alert system” drawing patient's attention to increasing stress levels or weight gain to optimize both psoriasis treatment and lifestyle to prevent an acute flare. Given that IMPROVE 2.0 is able to not only monitor psoriasis severity but also serve as an individualized feedback system for each patient, positive effects on psoriasis and overall healthy way of life can be expected and investigated by future randomized, controlled trials.

Our study has some limitations. First, we investigated only a limited cohort of patients in a proof-of-concept approach. Due to the small study cohort, a more advanced data analysis including robust statistical tests for significance of factors influencing future PASI changes was not possible. Second, IMPROVE 1.0 was only compatible to Android smartphones and many psoriasis patients were unwilling to participate due to technical difficulties in handling a smartphone app in general. Though both might lead to a selection bias, these observations are important to estimate psoriasis patients' willingness to use digital tools in real life and demonstrate that an app can only support a subgroup of patients with technical affinity. Furthermore, improvement of PASI scores during study participation must be interpreted with caution as they might be influenced by frequent physician consultation based on the study design. Despite these limitations, the strength of our study is that patients were followed up closely in a prospective manner over a period of 12 months. By comparing app performance to the current gold standard, namely, dermatologist's examination and collecting patient's feedback, this study proves that smartphone apps can be an important future component of a comprehensive therapeutic approach.

In summary, we demonstrated that a smartphone app is a valuable and reliable tool to assess disease activity, life quality, and potential influencing factors in psoriasis patients. This tool can markedly improve doctor–patient communication, facilitate interdisciplinary collaboration, and, most importantly, assign the patients an active role in disease management. Of note, we assessed temporal interrelations between PASI increase and lifestyle factors in individual patients. This points toward a high value of automated feedback loops—for example, by virtual disease managers helping patients to control individual risk factors—to improve disease activity in a future IMPROVE 2.0 app. These data are essential for the future implementation of digital tools in clinical routine care of complex, inflammatory diseases.

## Data Availability Statement

The original contributions presented in the study are included in the article/Supplementary Material, further inquiries can be directed to the corresponding author/s.

## Ethics Statement

The studies involving human participants were reviewed and approved by the Ethics Committee of the Faculty of Medicine of the Technical University of Munich (63/16S). The patients/participants provided their written informed consent to participate in this study.

## Author Contributions

NG-S and FL designed the study and wrote the paper. NG-S, SB, VB, and FL generated the data. NG-S and SB analyzed the data. SS programmed the app. TB revised the manuscript. All authors contributed to the article and approved the submitted version.

## Conflict of Interest

NG-S received speaker or consultant fees by LEO Pharma, Abbvie, Novartis and Lipidor AB. FL received speaker or consultant fees by Lilly, Amgen, Almirall, LEO Pharma, Abbvie, Roche, Sanofi, Janssen, Novartis not related to this manuscript. The remaining authors declare that the research was conducted in the absence of any commercial or financial relationships that could be construed as a potential conflict of interest.
