# Supplementary material for: IMPROVE 1.0: Individual Monitoring of Psoriasis Activity by Regular Online App Questionnaires and Outpatient Visits
Source: Front Med (Lausanne). 2021 Jun 22;8:648233. doi: 10.3389/fmed.2021.648233 (PMC8257945; doi:10.3389/fmed.2021.648233)
Supplement: Supplementary file 1 [file Data_Sheet_1.docx]

Supplementary Material

IMPROVE 1.0: Individual monitoring of psoriasis activity by regular online app questionnaires and outpatient visits

^1,2^Natalie Garzorz-Stark^∇,*^, MD, PhD, ^1^Sarah Beicht, MD^∇^, ^1^Veronika Baghin, MD, ^1^Sebastian P Stark, PhD, ^1^Tilo Biedermann, MD, ^1^Felix Lauffer, MD, PhD

^1^Technical University of Munich, Department of Dermatology and Allergy, Munich, Germany

^2^Division of Dermatology and Venereology, Department of Medicine Solna, and Center for molecular medicine, Karolinska Institutet; Stockholm, Sweden

∇=both authors contributed equally

*** Correspondence:**Natalie Garzorz-Stark
[natalie.garzorz@tum.de](mailto:natalie.garzorz@tum.de)


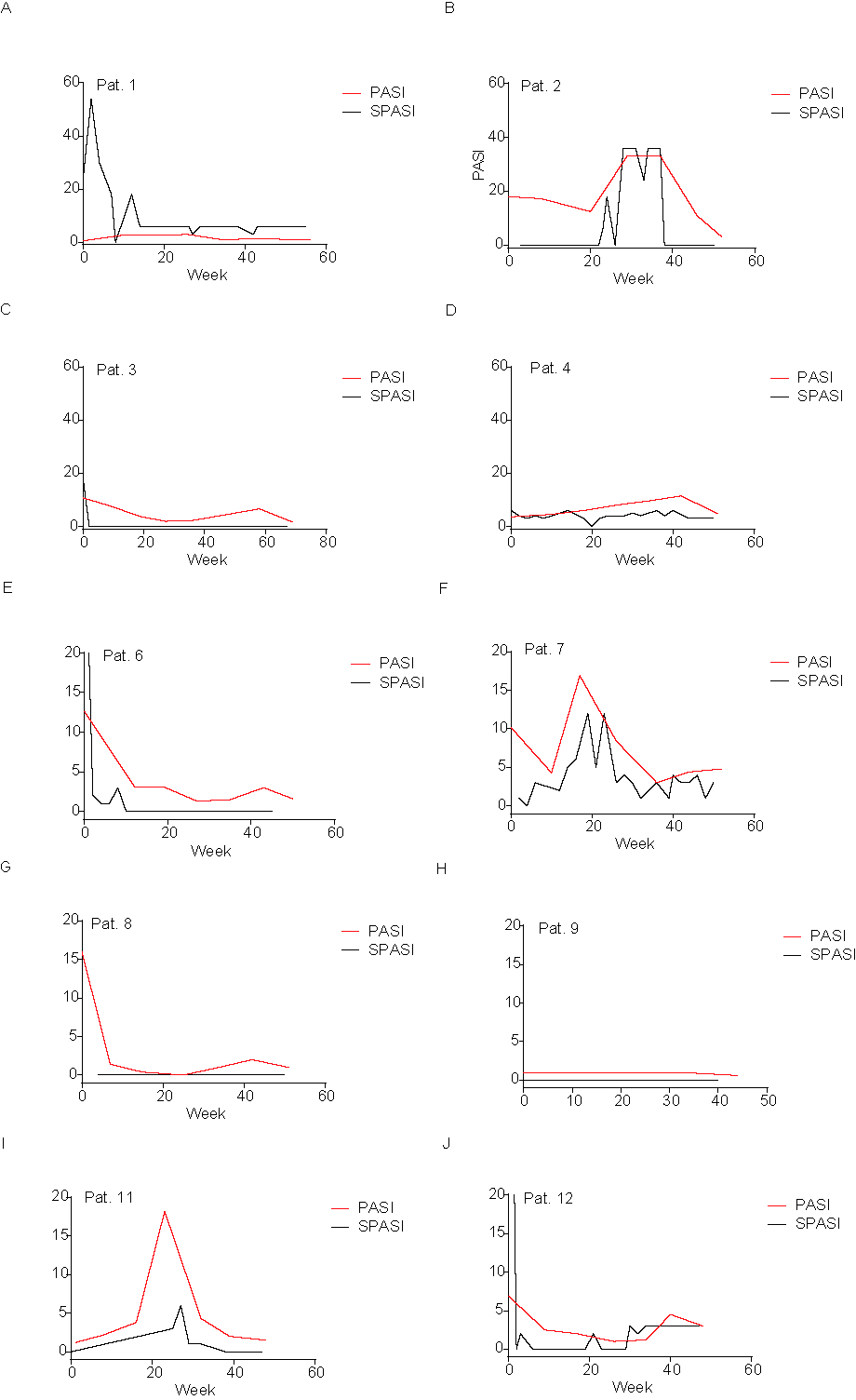


**Supplementary Figure 1**: Curves of PASI and SPASI of remaining patients of the study.

**
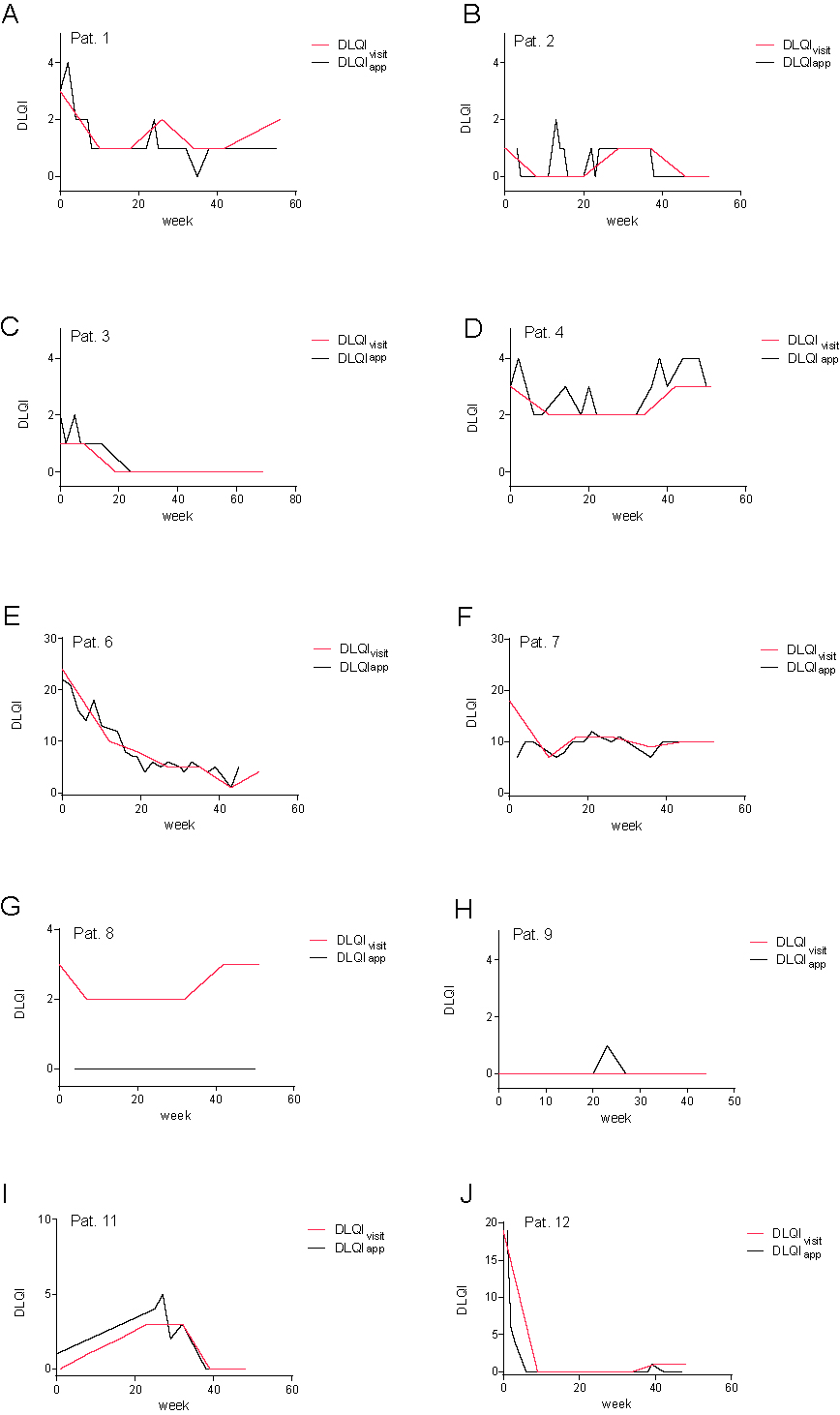
**

**Supplementary Figure 2**: Curves of DLQI_app_ and DLQI_visit_ of remaining patients of the study.


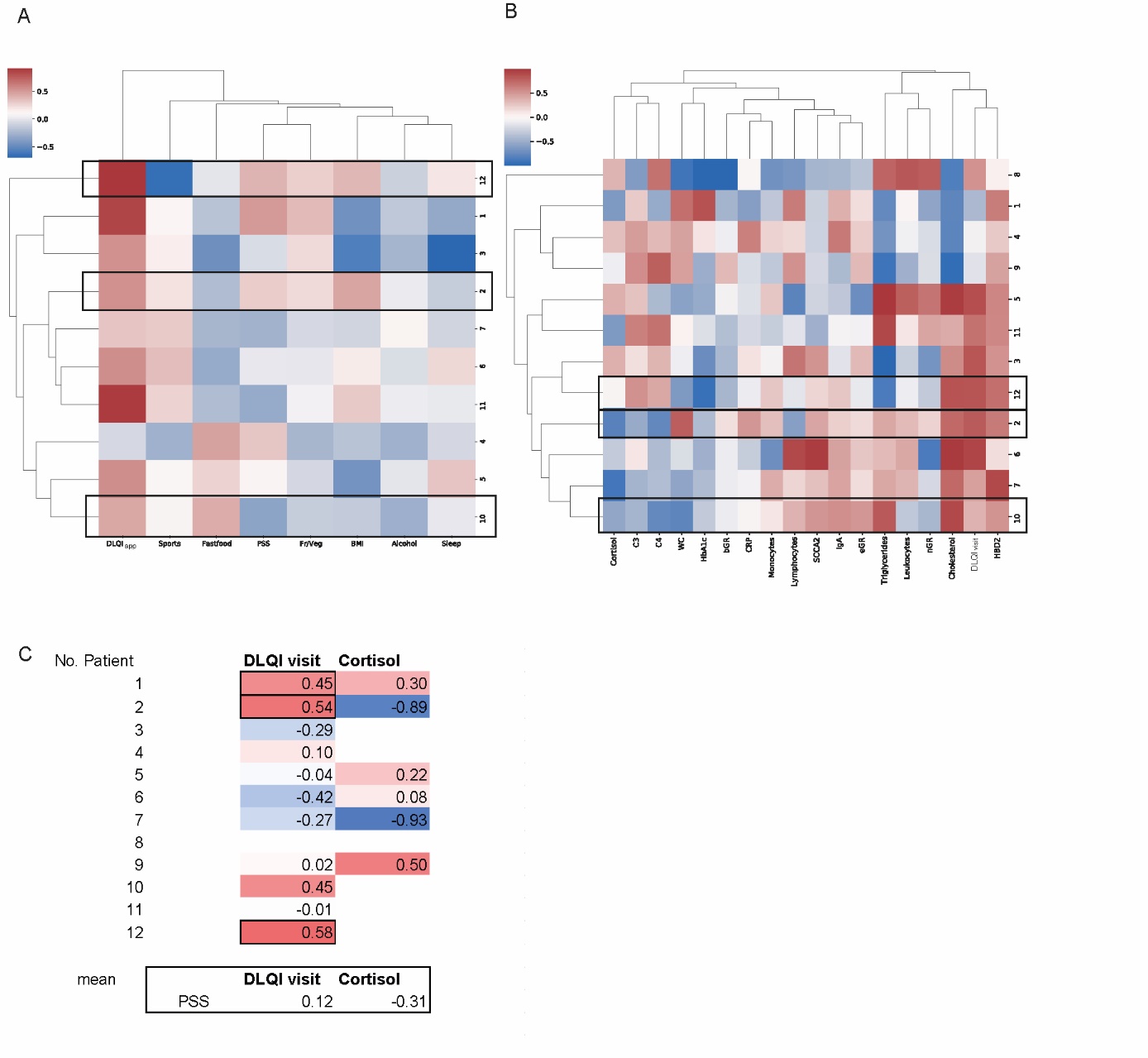


**Supplementary Figure 3**: Correlation matrix of SPASI (A) or PASI (B) with parameters assessed during the study. Patients shown in Figure 3 concerning stress curves are shown in black frames. Correlation coefficients of PSS and DLQI_visit_ as well as PSS and cortisol for all patients with significant correlations (p < 0.001) in small black frames. Mean correlation coefficient in big black frame.


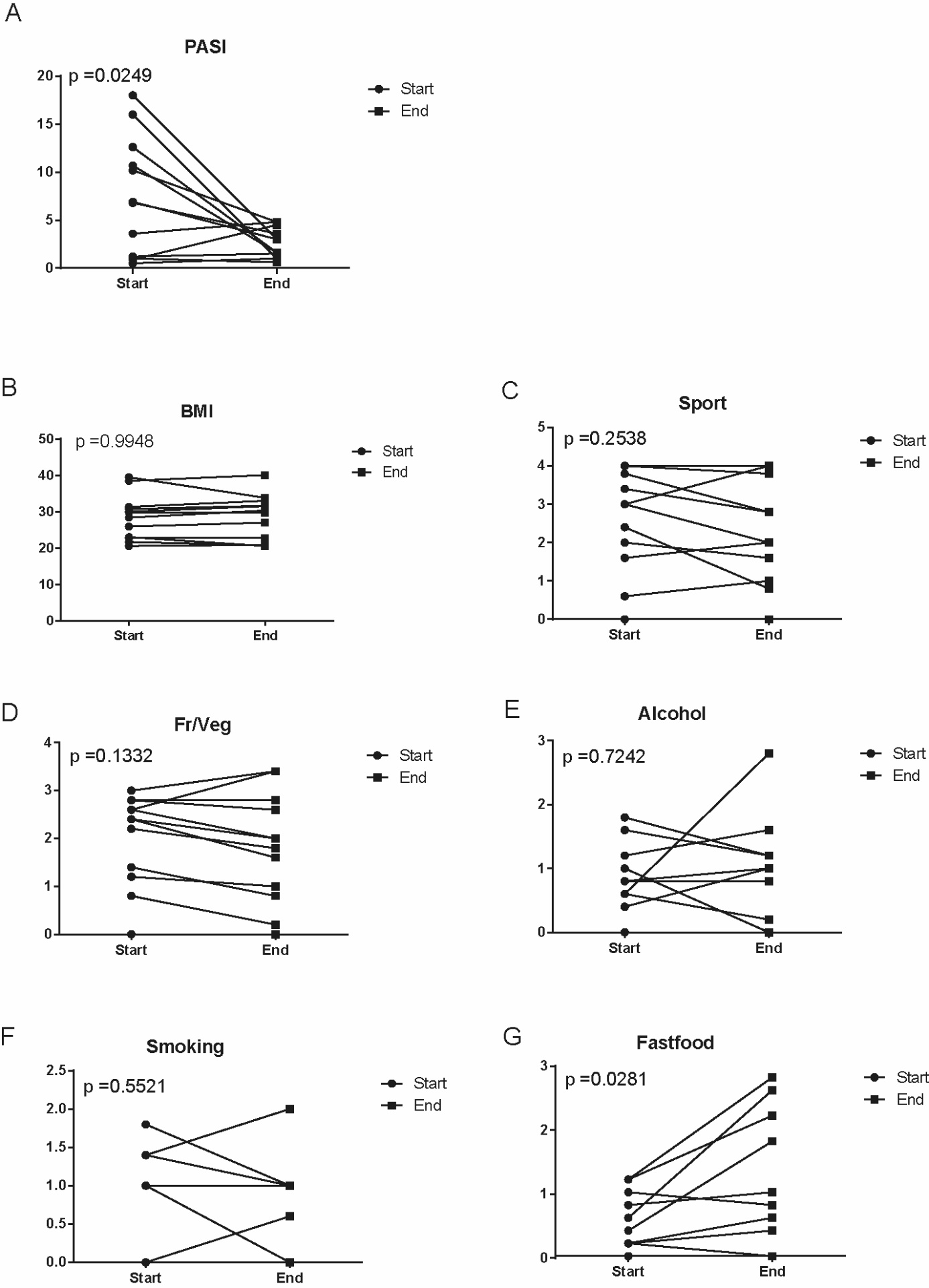


**Supplementary Figure 4**: Pairwise comparison of parameters at the start and at the end of the study for all patients. Fr/Veg = Consumption of fruits and vegetables
